# Dietary Polyphenols-Gut Microbiota Interactions: Intervention Strategies and Metabolic Regulation for Intestinal Diseases

**DOI:** 10.3390/biology14121705

**Published:** 2025-11-30

**Authors:** Huangkun Li, Ji’an Gao, Wenting Peng, Xihan Sun, Wentao Qi, Yong Wang

**Affiliations:** 1 School of Health Science and Engineering, University of Shanghai for Science and Technology, Shanghai 200093, China; 2 Academy of National Food and Strategic Reserves Administration, Beijing 100037, China

**Keywords:** polyphenols, gut microbiota, intestinal disease, bioavailability, bidirectional regulatory mechanism

## Abstract

In the exploration of the relationship between nutrition and health, dietary polyphenols, as bioactive substances derived from natural plants, have attracted much attention due to their diverse functional properties. Their health effects are highly dependent on the metabolic transformation by intestinal flora. The complex interaction between polyphenols and gut microbiota, as well as their role in the prevention and treatment of intestinal diseases, has become a research hotspot in the fields of nutrition and medicine. This review systematically explains the sources and classifications of polyphenols, points out their natural characteristic of low bioavailability. It also explores their intervention effects on intestinal diseases, analyzes the bidirectional action mechanism between polyphenols and gut microbiota, and elaborates on the specific pathways through which polyphenols improve intestinal diseases by regulating gut microbiota. Collectively, this review provides critical theoretical basis on polyphenols improving intestinal diseases for future research and clinical applications.

## Simple Summary

Dietary polyphenols are extensively sourced and can be classified in various ways, but they exhibit low bioavailability in humans. Meanwhile, polyphenols can utilize the interaction with gut microbiota to actively intervene in a variety of intestinal and extra intestinal diseases. Therefore, we described the existing knowledge regarding the classification of polyphenols as well as their bioavailability in this review. Furthermore, we discussed the specific action mechanisms of polyphenols on intestinal diseases, summarized the associations among polyphenols, immune cells, intestinal barrier and central nervous system signaling pathways, and put forward the problems that need to be solved in this field in the future.

## 1. Introduction

Polyphenols are abundantly present in plant-derived foods, including whole grain cereals, vegetables, fruits, tea and wine. Based on their chemical structures, they are primarily classified into flavonoids, phenolic acids, lignans, and stilbenes. In recent years, evidence has indicated that polyphenol intake may confer benefits to human health [1]. For instance, anthocyanin-rich red fruit juices have demonstrated potential to improve DNA integrity and influence lipid metabolism in human subjects [2]. Concurrently, polyphenol consumption exhibits preventive effects against cardiovascular diseases [3] and aging [4]. Therefore, polyphenols have received great attention. Nowadays, with the improvement of living standards, people are paying more attention to the nutritional components in their diet and adopting new dietary habits. It is of great significance to explore the potential mechanisms of polyphenols on human health.

The intestine, particularly the colon, is not only a site for digestion and absorption but also the body’s largest and most complex micro-ecosystem and a major immune organ [5]. Disruption of intestinal homeostasis is closely associated with the pathogenesis and progression of various intestinal disorders, as well as extra-intestinal disorders (autoimmune diseases, cancers, etc.) [6]. These include highly prevalent functional disorders such as irritable bowel syndrome (IBS) [7], severe chronic inflammatory diseases like inflammatory bowel disease (IBD) [8] and lethal malignancies such as colorectal cancer (CRC) [9].These conditions are often characterized by gut microbiota dysbiosis [10], impaired intestinal barrier function [11], aberrant immune responses [12] and dysregulation of gut–brain axis communication [13]. Apparently, gut health is of crucial importance to the human body. Research has found that there is a complex two-way interaction between polyphenols and gut microbiota. Polyphenols can effectively maintain intestinal homeostasis by regulating the composition of the microbiota and secondary metabolites. The microbiota can promote the biotransformation of polyphenols and improve their bioavailability.

Currently, numerous studies have reported the effects of dietary polyphenols on intestinal diseases. However, there are obstacles to understanding the mechanism of action between polyphenols and the gut microbiota. This review introduces the main sources, classifications and characteristics of bioavailability of dietary polyphenols. It comprehensively elaborates on how polyphenols improve intestinal-related diseases and exert gut protective effects through a bidirectional regulatory mechanism with the gut microbiota. The review focuses on elucidating the mechanisms of action of polyphenols, gut microbiota, and their derivatives on the intestinal barrier, immune system, and gut–brain axis. This review will provide a comprehensive perspective for understanding the role of dietary polyphenols as “gut microbiota modulators” in maintaining intestinal health and preventing and treating intestinal diseases.

## 2. Polyphenols in the Diet

### 2.1. Classification and Source of Dietary Polyphenols

**Figure 1:**
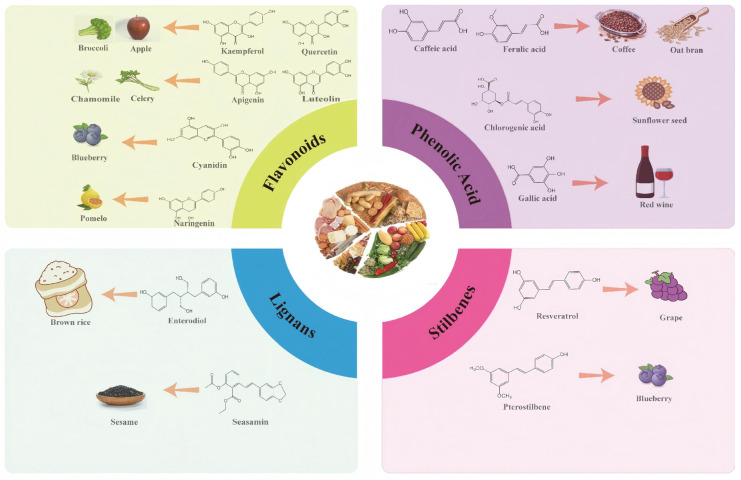
Classification and Sources of Polyphenols.

Polyphenols are a broad class of plant-based compounds characterized by multiple phenolic hydroxyl groups, which provide various health promoting effects. When considering dietary quality and nutritional value, the stability of polyphenols is of paramount importance. Without considering external interference factors, the stability of flavonoids follows this order: resorcinol type > catechin type > pyrogallol type [14]. The bioaccessibility of hydroxybenzoic acid during gastrointestinal digestion decreases with increasing numbers of hydroxyl substituents [15]. Consequently, different polyphenolic structures exhibit varying degrees of stability. Their types and concentrations vary significantly among different dietary sources, affecting their biological activity in the body. Based on chemical structure, polyphenols are classified into flavonoids, phenolic acids, lignans and stilbenes [16]. The representative chemical structures and dietary sources of these polyphenols are summarized in Figure 1.

Flavonoids, a major subclass of secondary metabolites, have a core structure made of two benzene rings connected by a three-carbon chain. Flavonoids are further divided into seven subclasses depending on their substitution patterns (flavonols, flavones, isoflavones, anthocyanidins, flavanones, flavanols and chalcones) [17]. For instance, luteolin is commonly found in celery, lettuce, and spinach [18], while anthocyanins are abundant in blueberries, strawberries, and grapes [19]. Recently, these flavonoids have been increasingly recognized as vital dietary supplements for improving health and preventing disease.

Phenolic acids are primarily divided into two groups: hydroxybenzoic acids with a C6-C1 structure, such as gallic, protocatechuic, and vanillic acids; and hydroxycinnamic acids with a C6-C3 structure, including caffeic, ferulic, and *p*-coumaric acids [20]. In the diet, hydroxybenzoic acids exist in various forms, including both free and bound states, and hydroxycinnamic acids predominantly exist in bound rather than free forms. For example, ferulic acid is often bound to arabinoxylans in cereal cell walls [21]. Hydroxycinnamic acids are generally more abundant and diverse than hydroxybenzoic acids [22], richly present in commonly consumed fruits, vegetables, and whole grains [23,24,25]. The structural stability and strong antioxidant properties of phenolic acids collectively underpin their health beneficial effects.

Plant lignans constitute a class of polyphenols synthesized from coniferyl alcohol precursors. They are abundant in fibre-rich dietary sources including seeds, grains, vegetables, and fruits [26]. Among these sources, flaxseed (*Linum usitatissimum*) contains exceptionally high levels of lignans, which is localized in the secondary walls of sclerenchyma cells within the seed coat [27]. Numerous studies have demonstrated that lignans possess a range of biological activities, with notable anti-cancer effects [28].

Stilbenes feature a C6-C2-C6 skeleton and originate from a more limited set of dietary sources, including grape wine, peanuts, and certain berries. The most extensively studied stilbene is resveratrol [29], which exists in several isomeric forms. Among these, trans-resveratrol exhibits more potent biological activity [30].

### 2.2. Bioavailability of Polyphenols

Dietary polyphenols have been extensively investigated for their diverse biological activities and potential in preventing chronic diseases. Bioavailability, which quantifies the proportion of a nutrient that enters systemic circulation, is dictated by its bioaccessibility and is crucially shaped by processes of digestion and absorption. Moreover, it is invariably influenced by co-ingested dietary compounds [31]. The journey of dietary polyphenols in the human body involves a multi-stage process of absorption, metabolism, and excretion. Initial absorption occurs partially in the stomach, with specific compounds like catechins, flavanols, and flavones being absorbed in the small intestine [32]. The bioavailability of dietary phenolic compounds can be enhanced by processing techniques including heating, crushing, and steaming [33]. In the gastrointestinal tract, polyphenols can be hydrolyzed by glucosidases, releasing highly lipophilic aglycones that diffuse into intestinal epithelial cells, improving their utilization [34]. Within these cells, aglycones are further enzymatic metabolized, leading to glucuronidation, methylation, or sulfation [35,36]. The resulting metabolites can either enter the bloodstream or be transported back into the intestinal lumen. Once flavonoids and their metabolites escape absorption in the small intestine, they proceed to the colon for extensive metabolism by gut microbiota into low-molecular-weight phenolic acids. This microbial transformation allows the phenolic acids to be absorbed across the colonic barrier much more effectively than the native compounds [37,38]. Certain polyphenols, such as flavonoid glycosides, are entirely dependent on this gut microbial metabolism to convert into absorbable aglycones or phenolic acids, enabling their entry into circulation [39]. Finally, excretion of polyphenols and their metabolites occur via urinary and fecal routes [40,41]. Although the overall processes of intake, absorption, metabolism, and excretion of polyphenols have been broadly characterized through long-term research, the bioavailability of specific polyphenol types remains variable (Table 1). Further research is essential to fully elucidate the role of gut microbiota in polyphenol metabolism.

## 3. The Impact of Polyphenols on Gut Diseases

### 3.1. Irritable Bowel Syndrome

IBS is a functional bowel disorder characterized by abdominal pain, bloating, and altered bowel habits [60]. Despite being non-fatal, the chronic and recurrent nature of the condition places a significant burden on the patient’s quality of life. Current pharmacological management primarily involves antidiarrheals, prokinetic agents, analgesics, and psychotropic drugs [61]. However, conventional therapies are often limited by suboptimal efficacy, significant adverse effects, and a narrow range of applicability. Consequently, there is a pressing need to develop novel and more effective treatment strategies.

Previous investigations have revealed the promising potential of polyphenols in the prophylaxis and treatment of IBS. Tsukasa Nozu et al. demonstrated that phlorizin mitigates visceral hypersensitivity, a crucial factor in IBS pathology, through inhibition of the SGLT2 and TLR4 signaling pathways [62]. Visceral hypersensitivity can be mediated by mast cell-derived mediators such as proteases and 5-hydroxytryptamine (5-HT). Addressing this mechanism, Qin et al. found that quercetin treats IBS through reducing colonic 5-HT content and decreasing enterochromaffin cell numbers [63]. Furthermore, restoring intestinal function in IBS requires repairing the damaged intestinal barrier. Puerarin promotes colonic epithelial cell proliferation by elevating the p-ERK/ERK ratio and strengthens the intestinal mucosal barrier by upregulating occludin expression [64]. The protective effect of luteolin, which reduces ROS levels and oxidative damage in colon tissue, attenuating excessive peristalsis and diarrhea, is mediated by the Nrf2/HO-1 signaling pathway [65]. Genistein ameliorates IBS-related dysmotility by enhancing c-kit expression in interstitial cells of Cajal [66]. This enhancement restores normal afferent signaling and smooth muscle contraction, thereby effectively alleviating diarrhea and improving intestinal function.

In addition to its gastrointestinal symptoms, IBS is often accompanied by emotional disturbances, particularly anxiety and depression. Resveratrol alleviates these psychological effects by reducing the expression of corticosterone and corticotropin-releasing hormone (CRH) in the HPA axis, while simultaneously increasing brain-derived neurotrophic factor (BDNF) and modulating the Wnt/β-catenin pathway in the hippocampus [67]. Polyphenols further regulate brain function via complex interactions involving the gut microbiota, immune system, and neurotransmitter activity [68]. Specifically, flavonoids modulate the composition of the gut microbiota, stimulating it to produce metabolites such as short-chain fatty acids (SCFAs), γ-aminobutyric acid (GABA), and BDNF. Following their production, certain metabolites undergo biotransformation into active neurotransmitters [69]. These bioactive compounds can produce anxiolytic effects, alleviate intestinal motility disorders, and reduce visceral hypersensitivity by targeting the PKA-CREB-BDNF and TLR4/MyD88/NF-κB signaling pathways [64,70]. Collectively, this evidence demonstrates that polyphenols can counteract the multifaceted pathophysiology of IBS through the gut–brain axis.

### 3.2. Inflammatory Bowel Disease

IBD is a chronic inflammatory disorder, broadly classified into ulcerative colitis and Crohn’s disease according to the location and severity of the lesions [71]. Its primary clinical manifestations are diarrhea, abdominal distension, pain, and even bloody stool. The pathogenesis of IBD is multifactorial, involving genetic predisposition, environmental triggers, dysregulated immune responses, and gut microbiota dysbiosis [72]. Current therapeutic strategies include non-targeted approaches, such as aminosalicylates, glucocorticoids, and immunomodulators [73,74]; and targeted biological therapies, including anti-TNF, anti-IL-12/IL-23, and anti-α4β7 integrin agents [75,76,77]. However, biological therapies fail to produce a response in approximately 30% of patients; moreover, a gradual diminution of efficacy is frequently observed over time [78].

Polyphenols ameliorate IBD symptoms and pathology primarily through their anti-inflammatory and immunomodulatory effects [79]. It has been shown that thyme polyphenols ameliorate ulcerative colitis via inhibition of the TLR4/NF-κB-NLRP3 inflammasome pathway [80]. Additionally, mango polyphenols attenuate IBD by suppressing neutrophil infiltration and reducing biomarkers of inflammation including interleukin-8, growth-regulated oncogene and granulocyte macrophage colony-stimulating factor [81]. In a mouse model of IBD, green tea polyphenols suppress the innate immune response in the colon through the modulation of STAT1 and PPARα/γ pathways [82]. Chlorogenic acid ameliorates colitis by impairing M1 macrophage polarization through the inhibition of Pkm2 and NLRP3 [83]. In contrast, ferulic acid acts via a neutrophil-dependent mechanism, which involves suppressing the formation of neutrophil extracellular traps. Thus, the therapeutic potential of polyphenols in IBD arises from their capacity to suppress inflammatory mediators and modulate key immune cell functions.

### 3.3. Colorectal Cancer

CRC is a highly prevalent malignancy of the digestive system. It ranks as the second leading cause of cancer-related mortality worldwide [84]. A major challenge in its treatment is the frequent development of drug resistance, which often limits the efficacy of conventional anticancer therapies [85]. Therefore, it remains crucial to elucidate the underlying mechanisms of CRC progression, and to identify novel therapeutic targets.

Given their favorable safety profile and bioactive properties, polyphenols have emerged as promising agents with significant antitumor activity against CRC. In vitro studies using the HT29 colorectal cancer cell line showed that isoeugenol-based phenolic compounds suppressed cancer cell proliferation and migration and induced apoptosis by inhibiting the expression of MMP-2, MMP-9, VEGF, and HIF-1α [86]. Gallotannin exerts its anticancer effects in colon cancer by inhibiting the JAK/STAT pathway [87]. In addition, polyphenols exert antitumor effects by inducing cell cycle arrest in colon cancer cells. Studies have shown that exposure to non-extractable polyphenols from cranberries induces cell cycle arrest in the G0/G1 phases, resulting in significant cellular apoptosis in colon cancer cells [88]. Moreover, resveratrol induces G0/G1 phase cell cycle arrest through the downregulation of cyclin D1, cyclin-dependent kinase (CDK)4, and CDK6 [89]. The primary mechanism by which polyphenols alleviate colon cancer is through the regulation of target genes, which impairs critical cellular processes such as proliferation, differentiation, apoptosis, and cell cycle progression.

## 4. Bidirectional Relationship Between Polyphenols and Gut Microbiota

### 4.1. The Modulatory Effects of Polyphenols on Intestinal Microbiota Composition

The human body is host to a community of 10 to 100 trillion microorganisms, collectively known as the microbiome, which are crucial for maintaining health [90,91]. This article discusses bacteria but not fungi or viruses. At the phylum level, the intestinal microbiota is dominated by the Bacteroidetes, Firmicutes, Actinobacteria, Proteobacteria, and Verrucomicrobia [92,93]. Bacteroidetes and Firmicutes are generally the dominant gut phyla, comprising over 90% of the total intestinal bacterial population. A gradient of increasing microbial abundance exists along the gastrointestinal tract, characterized by lower proportions in the stomach and the highest density in the colon [94]. The intestinal microbiota is essential for the maintenance of the intestinal mucosal barrier, protection from pathogenic invaders, and homeostatic regulation of the immune system. A disruption in the microbial composition, known as dysbiosis, is associated with the onset of diseases such as obesity and type 2 diabetes [95].

Research has demonstrated that polyphenols or polyphenol-rich foods can alter the composition of the gut microbiota (Table 2) [96]. Their regulatory effects are exerted primarily through two key mechanisms. On the one hand, increasing the abundance of beneficial bacteria and restoring gut homeostasis can prevent and intervene in diseases [97]. Polyphenols act as prebiotics by selectively promoting the growth of beneficial bacteria such as *Lactobacillus* and *Bifidobacterium* [98]. Meanwhile, the increased abundance of beneficial bacteria indirectly suppresses pathogenic strains such as *Escherichia coli* [99], *Salmonella* [100], and *Helicobacter pylori* [101]. Beyond their direct effects, certain polyphenols such as resveratrol modulate the surface architecture of probiotic bacteria, thereby enhancing their adhesion to the mucosal layer and promoting microbial colonization [102]. Additionally, as Proteobacteria contain numerous pathogenic genera, they may contribute to gastrointestinal inflammation and thereby predispose humans to metabolic diseases [103]. Curcumin significantly increases the abundance of bacterial genera such as *Bacteroides*, *Akkermansia*, *Parabacteroides*, *Alistipes*, and *Alloprevotella*. This modulation of gut microbiota is associated with the alleviated metabolic features of hepatic steatosis and insulin resistance [104].

On the other hand, beyond influencing individual bacteria, polyphenols can also alter the composition of the gut microbiota by changing the ratios between different genera. This promotes a more balanced and healthier microbial community composition. At the phylum level, the major bacterial groups primarily involved are Firmicutes and Bacteroidetes. An altered F/B ratio is regarded as an indicator of dysbiosis relevant to metabolic diseases [105]. Inducing gut microbiota dysbiosis with clindamycin hydrochloride, apple polyphenols administered at three doses reduced the Firmicutes-to-Bacteroidetes (F/B) ratio from 0.89 to 0.77, 0.36, and 0.41, indicating gradual changes in gut microbiota composition under polyphenols intervention [106]. Studies have demonstrated an imbalance in the gut microbiota of individuals with hyperlipidemia, which is manifested as an increased F/B ratio and a diminished abundance of beneficial bacteria [107]. Intervention with tea polyphenols was observed to significantly suppress weight gain in hyperlipidemic rats, improve metabolic indices such as blood glucose and lipids, restore the F/B ratio to normal levels, and enhance intestinal health. At the genus level, gallic acid, *p*-coumaric acid, and phlorizin increase the abundance of several SCFA-producing bacteria, such as *Odoribacter*, *Muribaculum*, *Alistipes*, *Lactococcus*, *Unspecified_Ruminococcaceae*, and *Turicibacter* [108,109,110]. This leads to elevated levels of butyrate and acetate, which help strengthen the intestinal barrier and reduce inflammation [111,112].

**Table 2:** Modulatory effect of polyphenols on gut microbiota.

Classification	Polyphenols	Model	Dose	Related microbiota	Reference
Flavonoids	Epigallocatechin-3-gallate	Colitis	50 mg/kg	↑*Akkermansia* and *Lactococcus*	[113]
	Apigenin	Visceral hypersensitivity	20 mg/kg	↑*Muribaculaceae* and *Limosilactobacillus* ↓*Escherichia-Shigella* and *Enterococcus*	[114]
	Quercetin	Metabolic syndrome	50 mg/kg	↑*Jeotgalicoccus* and *Corynebacterium_1* ↓*Alloprevotella* and *Ruminiclostridium_9*	[115]
	Kaempferol	Periodontitis	1 mg/kg	↑*Ruminococcus* and *Turicibacter* ↓*Ligilactobacillus* and *Bifidobacterium*	[116]
	Daidzein	Chronic restraint stress	10 mg/kg 20 mg/kg 40 mg/kg	↑Verrucomicrobiota and Campilobacterota ↓Actinobacteriota	[117]
	Naringenin	Colorectal cancer associated with a high-fat diet	100 mg/kg	↑*Intestinimonas* and *Parabacteroides* ↓Bifidobacteriales and Coriobacteriia	[118]
	Procyanidin	Intestinal barrier dysfunction	200 mg/kg	↑Lachnospiraceae and Bacteroidaceae ↓*Ruminococcus_1* and *Bacteroidales S24-7*	[119]
	Cyanidin	Natural aging	50 mg/kg	↑*Faecalibaculum* and *Bifidobacterium*↓*Ligilactobacillus* and *Desulfovibrionaceae*	[4]
Phenolic acids	Vanillic Acid	Ulcerative Colitis	100 mg/kg 200 mg/kg 400 mg/kg	↑*Ligilactobacillus* ↓*Alistipes* and *Bacteroides*	[120]
	Caffeic acid	Intestinal injury	500 mg/kg	↑*Alloprevotella* and *[Eubacterium]_coprostanoligenes_group* ↓*Prevotella*	[121]
	Ferulic acid	Diabetic syndrome	30 mg/kg	↑Lachnospiraceae and Bacteroidaceae	[122]
	Chlorogenic Acid	Colon mucosal damage induced by a high-fat diet	100 mg/kg	↑Chlorophyta and Tenericutes ↓Elusimicrobia	[123]
Lignans	Sesamol	DSS-Induced colitis	100 mg/kg	↑Odoribacter and *Butyricicoccus*	[124]
	Pinoresinol	Ovariectomy-induced osteoporosis	5 mg/kg	↑*Akkermansia* and *Lachnospiraceae_NK4A136* ↓*Lactobacillus* and *Prevotella*	[125]
Stilbenes	Resveratrol	Traumatic spinal cord	200 mg/kg	↑Lactobacillales and *Lactobacillus*	[126]
	Pterostilbene	Osteoarthritis	200 mg/kg	↑*Alistipes indistinctus* and *Butyricicoccus pullicecorum* ↓Clostridium symbiosum and Marvinbryantia formatexigens	[127]

The upward arrow in the table indicates the increase in microbial abundance, while the downward arrow indicates its decrease.

### 4.2. Impact of Polyphenols on Gut Microbiota Metabolites

**Figure 2:**
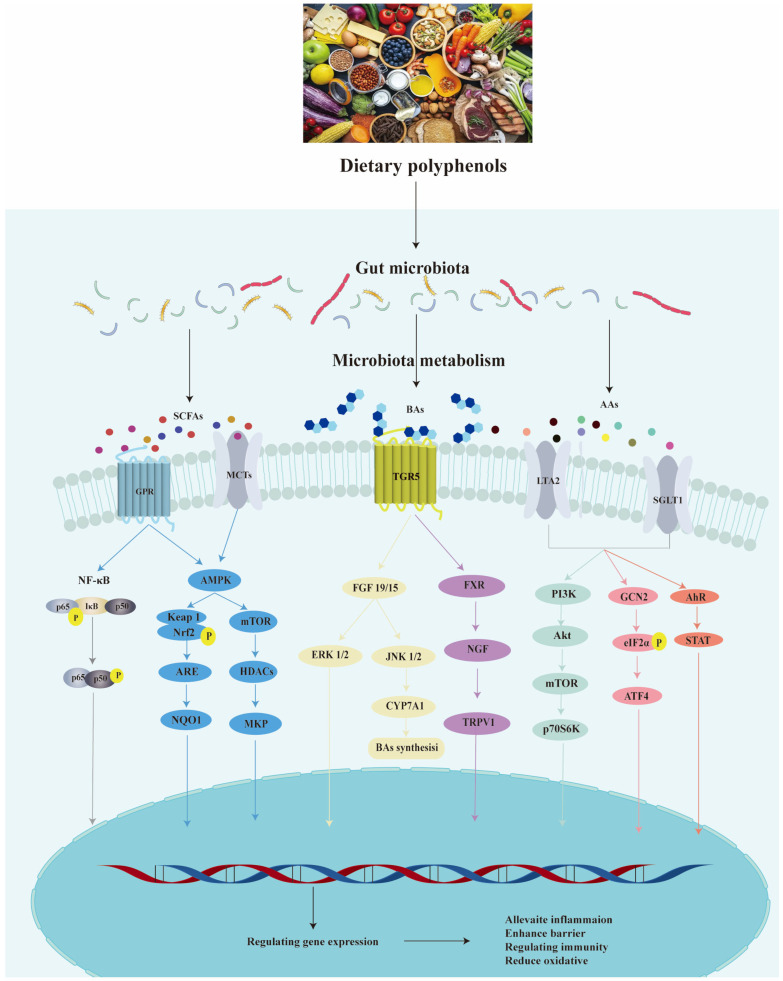
Dietary polyphenols modulate the gut microbiota, leading to the production of metabolites that regulate key signaling pathways. Polyphenols alleviate inflammation and cell apoptosis by promoting the microbial production of SCFAs, which subsequently modulate the NF-κB and AMPK signaling pathways. BAs act through the FGF and FXR signaling pathways to attenuate intestinal barrier impairment and pain signal transmission, while also regulating BA synthesis. Metabolites derived from aromatic AAs can mitigate stress-induced damage and immune responses by engaging the PI3K, GCN2, and AhR signaling pathways. Collectively, these mechanisms contribute to the restoration of intestinal health and the maintenance of homeostasis.

Current understanding indicates that polyphenols modulate the gut microbiota, producing metabolites such as SCFAs, bile acids (BAs), and amino acids (AAs) [128]. These metabolites bind to specific receptors, activate intestinal cells, and thereby exert beneficial effects on gut health (Figure 2). SCFAs, such as acetate, propionate, and butyrate, are primarily derived from the microbial fermentation of dietary fiber in the gut [129]. A study demonstrated that apple polyphenols increased the concentrations of total SCFAs during gut microbiota fermentation, from 2.271 ± 0.029 mM to 37.093 ± 0.478 mM over 24 h [130]. Epigallocatechin-3-gallate (EGCG) was found to utilize specific gut microbiota, including *Lactobacillus*, *Ruminococcus*, *Clostridium*, and *Akkermansia*, to elevate total fecal SCFAs from 5.40 ± 0.56 μg/mg to 6.88 ± 0.54 μg/mg. This increase was reflected in the individual levels of acetate (from 4.75 ± 0.15 to 5.11 ± 0.14 μg/mg), propionate (from 0.28 ± 0.07 to 0.62 ± 0.06 μg/mg), and butyrate (from 0.34 ± 0.11 to 1.04 ± 0.27 μg/mg), thereby ameliorating non-alcoholic fatty liver disease and endotoxemia [131]. Acetic acid is the principal end product of intestinal glycolysis, whereas butyrate is primarily produced by specific bacterial families, including Lachnospiraceae and Ruminococcaceae [132]. It has been shown that blackberry anthocyanins increase acetic acid levels, leading to notable improvements in lipid metabolism and alleviation of liver injury [133]. Distiller’s grain polyphenols increase the abundance of beneficial gut microbiota, such as *Bifidobacterium*, *Ruminococcus*, *Lactobacillus*, and *Akkermansia*, which consequently elevates acetate production [134]. Propionic acid, a major fermentation product of *Bacteroides*, is absorbed into the bloodstream and transported to the liver for further breakdown and metabolism. In the liver, it regulates the conversion of pyruvate to glucose and suppresses lipogenesis [135]. It also binds to GPR41 and thereby induces lipolysis via the PKA–PPARα cascade, contributing to anti-obesity and anti-steatotic effects. Butyric acid, which is mainly produced by Firmicutes, acts as the principal energy source for colonocytes and contributes to the maintenance of intestinal barrier integrity through the regulation of key tight junction proteins, including claudin-1, occludin, and ZO-1 [136]. EGCG stimulates butyrate production by restructuring the gut microbiota [113]. Punicalagin increases the butyrate-producing bacterial groups *Eubacterium_coprostanoligenes_group* and *Lachnospiraceae* in diabetic mice. Correlation analysis indicates that *Eubacterium_coprostanoligenes_group* exhibits negative correlations with triglyceride and blood glucose levels [137].

BAs are amphipathic steroids synthesized in the liver as primary BAs. Upon entry into the intestinal environment, primary BAs are metabolized by the gut microbiota into secondary BAs through a series of reactions that include deconjugation, dehydrogenation, and dihydroxylation [138,139]. This transformation depends on microbial bile salt hydrolase (BSH). Apple polyphenols enhance secondary BA metabolism by modulating BSH-rich microbiota such as *Lactobacillus* and *Bifidobacterium* [140]. The resulting secondary BAs can indirectly activate the Farnesoid X receptor (FXR) and Takeda G protein-coupled receptor 5 (TGR5), leading to increased transcriptional expression of cytochrome P450 7A1 (CYP7A1). Grain-derived flavonoids reduce the abundance of lipid metabolism-associated microbiota (*Lachnoclostridium*, *Blautia*, *Lachnospiraceae_UCG-006*, *Roseburia*, and *Faecalibaculum*) [141]. This microbial shift activates the FXR signaling pathway, leading to the regulation of CYP7A1 and the upregulation of the major BAs transporters NTCP and BSEP, which are two essential transporters for uptake and excretion of hepatic BAs. Furthermore, chokeberry polyphenol downregulate lipid synthesis factors, including PPARγ, UCP1, and PGC-1α [142]. This effect is likely mediated through the activation of the TGR5 signaling pathway by specific gut bacteria (*Bacteroides*, *Prevotella*, *Clostridium*, *Eubacterium*, and *Ruminococcaceae*), which concurrently modulates BA composition by decreasing cholic acid and deoxycholic acid levels while increasing chenodeoxycholic acid content. Collectively, these findings reveal that polyphenols regulate BA metabolism and attenuate metabolic disease progression primarily through microbiota-dependent mechanisms, strategically leveraging the FXR and TGR5 pathways.

As common gut microbiota-derived metabolites, branched-chain amino acids (BCAAs, e.g., leucine, valine, and isoleucine) and aromatic amino acids (AAAs, e.g., tyrosine, phenylalanine, and tryptophan) play critical roles in host physiology. Elevated circulating BCAA levels are recognized as a metabolic hallmark of obesity, insulin resistance, dyslipidemia, non-alcoholic fatty liver disease, and type 2 diabetes [143,144]. Furthermore, BCAA metabolism can exacerbate intestinal inflammation via the mTOR/p70S6K signaling pathway. Bergenin mitigates this inflammation by reducing the *Bacteroides vulgatus* abundance and correspondingly lowering BCAA levels [145]. Pomegranate peel polyphenols can promote valine metabolism and reduce the levels of pro-inflammatory factors by increasing the abundance of *Roseburia* and *Christensenellaceae_R-7_group* and decreasing the abundance of *Blautia* [146]. As a precursor for the synthesis of monoamine neurotransmitters, AAA plays a crucial role in immune regulation, oxidative stress, and neuronal excitability. Among AAAs, tryptophan has the most complex and unique structure. The gut microbiota initiates a metabolic pathway for tryptophan by converting it into kynurenine, indole and its derivatives [147].Tea polyphenols promote phenylalanine and tryptophan metabolism by increasing the abundance of *norank_f__Muribaculaceae*, *Bifidobacterium*, and *Allobaculum*, and decreasing the abundance of *Helicobacter*, *Bacteroides*, and *Prevotellaceae UCG-001* [148]. Curcumin promotes tryptophan metabolism by increasing the abundance of *Lactobacilli* and may repair the intestinal epithelial barrier in inflammatory mice by activating the AhR pathway [149]. Consequently, while studies of gut microbial amino acid metabolites are currently dominated by BCAAs and AAAs, the contributions of other amino acids to gut health warrant continued attention.

### 4.3. The Impact of Intestinal Microbiota on Polyphenol Metabolism

Polyphenols can influence the intestinal flora and their derivatives, while the gut microbiota also plays a role in metabolizing polyphenols. The intestinal microbiota affects the stability of dietary polyphenols through enzymatic reactions, including deglycosylation, sulfation, glucuronidation, C-ring cleavage of the benzo-γ-pyrone system, dehydroxylation, decarboxylation, and hydrogenation [150,151,152,153]. Most O-glycosides are converted to aglycones [154], which are further conjugated with O-glucuronide and/or O-sulfate forms [155]. Then, the intestinal microbiota performs catabolic transformations, such as carbon-carbon cleavage of aromatic rings, decarboxylation, hydrogenation, and dehydroxylation of olefin moieties. For example, quercetin, under the catalysis of intestinal microorganisms, typically produces protocatechuic acid (3,4-dihydroxybenzoic acid) and 3,4-dihydroxyphenylacetic acid as its primary metabolites [156]. Then, they can be converted into 4-hydroxyphenylacetic acid and a one-carbon unit through dehydrogenation by intestinal bacteria [157]. Subsequently, these metabolites can be catalyzed by microbial methyltransferases to generate methylated derivatives such as homovanillic acid and 3-methoxy-4-hydroxyphenylacetic acid [158]. These compounds undergo decarboxylation to form *p*-cresol, followed by side-chain oxidation yielding benzoic acid derivatives, which are further metabolized to hippuric acid and excreted renally. The above results indicate that the gut microbiota promotes the metabolism of polyphenols through enzymatic reactions in multiple ways and improves the utilization rate of polyphenols.

## 5. Gut Microbiota-Mediated Amelioration of Intestinal Diseases by Polyphenols

### 5.1. Strengthen the Intestinal Barrier Function

The intestinal barrier is defined as a functional entity separating the gut lumen from the inner host. It consists of mechanical elements (mucus and epithelial layer), humoral elements (defensins and immunoglobulin A), immunological elements (lymphocytes and innate immune cells), and muscular and neurological elements [159]. Essential for maintaining intestinal barrier integrity, tight junction (TJ) proteins are composite molecular structures composed of multiple proteins, including occludin, claudins, and tricellulin [160]. It has been demonstrated that dietary polyphenols affect TJ expression and thus influence the intestinal barrier by shaping the gut microbiota [161,162]. Grape polyphenols mitigate inflammation-induced intestinal injury by enriching the abundance of *Akkermansia muciniphila*, upregulating Muc2 expression, increasing the number of ileal goblet cells, and enhancing mucus layer thickness [163]. Grape polyphenols suppress the abundance of pathogenic bacteria by modulating the proliferation of beneficial bacteria, specifically *Akkermansia* and *Lactobacillus*, and upregulate the mRNA expression of genes related to polyphenol absorption [164]. The expression levels of these key genes show a positive correlation with TJ proteins, thereby contributing to the restoration of intestinal barrier integrity. Turmeric nonextractable polyphenols enhance the abundance of *Alloprevotella* and *Ileibacterium*, thereby promoting butyrate production, preventing mucosal atrophy, and boosting TJ expression [165]. In addition, resveratrol and its microbial metabolites 3-(4-hydroxyphenyl)-propionic acid (4HPP) activate AMPK Pathway [166]. This action contributes to the maintenance of epithelial barrier function by modulating the expression of TJ proteins. Collectively, polyphenols preserve intestinal homeostasis by reinforcing the mucus layer and upregulating the TJ proteins.

### 5.2. Maintain Immune Homeostasis

Immune homeostasis refers to the balanced state established by the body between eliminating foreign pathogens and maintaining self-tolerance. The disruption of this balance is closely associated with the occurrence and development of various diseases, including autoimmune diseases, chronic inflammation, metabolic syndrome, and malignant tumors [167,168]. In recent years, plant-derived polyphenols have attracted considerable research attention due to their pleiotropic immunomodulatory effects and superior safety. Polyphenols can suppress excessive inflammatory responses and enhance immune defense capabilities. As core effector cells of the innate immune system, macrophages can polarize into pro-inflammatory M1 or anti-inflammatory M2 phenotypes in response to different microenvironmental cues [169]. *Forsythia suspensa* polyphenols promote beneficial bacteria such as *Bacteroidete* and *Allobaculum*, which concomitantly inhibits M1 polarization and promotes M2 anti-inflammatory macrophage populations [170]. Furthermore, *Rosa roxburghii tratt* polyphenols revealed a significant increase in SCFA-producing genera such as *Blautia*, *Bacteroides*, and *Roseburia* [171]. These polyphenols subsequently modulate the TLR signaling pathway by regulating intermediate metabolites of the tricarboxylic acid cycle, which in turn influences macrophage phagocytic activity and cytokine release.

Polyphenols influence the differentiation and activity of adaptive immune cells by modulating the function of innate immune cells. Besides macrophages, other innate immune cells are also important targets for the action of polyphenols. Neutrophils, serving as the “first line of defense” against pathogen invasion, can cause tissue damage when overactivated [172]. Studies demonstrate that ferulic acid increases the abundance of *Bifidobacterium pseudocatenulatum*, promoting butyrate production, which in turn inhibits the generation of pro-inflammatory mediators in neutrophils [173,174]. In the adaptive immune system, the differentiation direction of CD4^+^ T cells directly shape immune responses. Tannins and ellagic acid in pomegranate peel extract increase the abundance of *Prevotellaceae*, *Lachnospiraceae*, *Ruminococcaceae*, and *Lactobacillaceae*, inhibit the activation of microglia and macrophages, ameliorate the severity of experimental autoimmune encephalomyelitis in mice [175]. Moreover, polyphenols modulate the gut microbiota composition, and promote the differentiation of naive T cells into Th1 and regulatory T cells, while inhibiting the polarization of pro-inflammatory subsets including Th2 and Th17 [176]. The above findings reveal the molecular mechanism by which polyphenols regulate immune cell function through gut microbiota and their metabolites to maintain intestinal homeostasis.

### 5.3. Improve the Central Nervous System

The central nervous system (CNS) is one of the most complex systems in the human body, regulating essential physiological processes such as cognition, emotion, motor function, and homeostasis. In recent years, the gut–brain axis has been identified as a bidirectional communication network between the gut and the brain, mediated through neural, endocrine, immune, and metabolic pathways. Key components include the gut microbiota, intestinal mucosal barrier, vagus nerve, neurotransmitters, and immune factors [177,178]. Accumulating evidence indicates that the gut microbiota can modulate CNS activity, brain function, and host behavior via the gut–brain axis.

**Figure 3:**
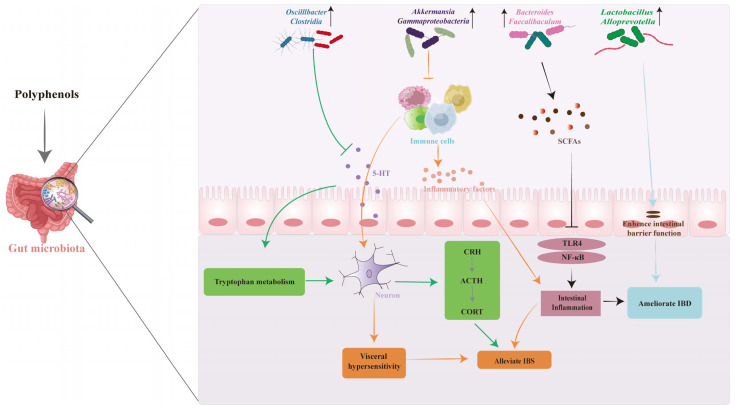
Polyphenols inhibit the release of inflammatory factors and 5-HT by immune cells, improve pain signal transmission of neurons and HPA axis to alleviate IBS through *Oscillibacter*, *Clostridia*, *Akkermansia*, and *Gammaproteobacteria*. Polyphenols promote the content of SCFAs and intestinal barrier integrity, inhibiting inflammatory pathways and enhancing intestinal function to alleviate IBD through *Bacteroides*, *Faecalibaculum*, *Lactobacillus*, and *Alloprevotella*.

Polyphenols exert neuroprotective properties by orchestrating compositional changes in the gut microbiota and modulating microbial metabolites. Oolong tea polyphenols (OTP) enhance the relative abundance of *Muribaculaceae* and *Clostridia_UCG-014*, while reducing that of *Desulfovibrio* [179]. Furthermore, OTP elevates BDNF levels and upregulates the expression of synaptic proteins postsynaptic density protein 95 and synaptophysin, which are critical for reinforcing synaptic connectivity. Through these mechanisms, OTP modulates synaptic plasticity and ultimately ameliorates cognitive impairment. EGCG promotes SCFA synthesis by increasing the abundance of *Alloprevotella*, *Muribaculaceae*, and *Bacteroides*, and inhibits PPAR-γ and NF-κB pathways, illustrating a microbiota-dependent mechanism of neuroregulation [180]. Parallelly, remodeling of the microbiome gut–brain axis represents a potential mechanism by which quercetin promotes neuroprotection in a repeated mild traumatic brain injury mouse model [181]. As a key neurotransmitter in the brain, 5-HT production can be promoted through a synergistic interaction between cyanidin-3-O-glucoside derivatives and the probiotic bacterium *Lactobacillus reuteri* LJJ 240337 [182]. Subsequently, 5-HT activates the cAMP/PKA/CREB signaling pathway, thereby contributing to neuroprotective effects. In addition, under stress conditions, CRH activates the hypothalamic–pituitary–adrenal (HPA) axis [183], resulting in elevated circulating glucocorticoid levels. This increase impairs the hippocampal region and induces neurotoxicity [184]. Polyphenols from *Hemerocallis citrina* Baroni have been shown to reduce the levels of CRH, ACTH, and CORT by restoring the abundance of *Monoglobus*, *unidentified_Clostridia*, and *Alloprevotella* [185]. This modulation helps to rebalance HPA axis activity and alleviates depressive-like behaviors in rats. In conclusion, polyphenols can ameliorate intestinal-related diseases by regulating microbiota metabolites, the immune nervous systems, and the intestinal barrier through gut microbiota (Figure 3).

## 6. Conclusions

Over the past decades, significant advances have been made in understanding the relationship between dietary polyphenols and gut health. Polyphenols have a health impact on the human body through their interaction with the gut microbiota. The interaction not only promotes the decomposition and metabolism of polyphenols, but also shapes the gut microbiota, increase the levels of short-chain fatty acids, bile acids and amino acids, enhances the function of the intestinal epithelial barrier, and strengthens the connection between the intestine and the central nervous system. This review summarizes the sources and structures of dietary polyphenols and explores how polyphenol–microbiota interactions ameliorate gut-related diseases by reinforcing the intestinal barrier, maintaining immune homeostasis, and modulating the gut–brain axis. In conclusion, the crosstalk between polyphenols and the gut microbiota is pivotal in managing intestinal diseases, suggesting that dietary polyphenols supplementation to modulate the gut microbiome may be an effective therapeutic strategy.

Despite considerable advances in our understanding of the relationship between polyphenols and gut microbiota, most current studies remain insufficient. For instance, many aspects of the microbial metabolism of polyphenols remain unclear, including which specific bacterial strains are involved, and which key enzymes are responsible for metabolizing particular polyphenols. It is also uncertain whether the effects are exerted by the parent polyphenols or their small-molecule metabolites. Their inherent structural diversity, interactions with the food matrix, and extensive gastrointestinal digestion and metabolism result in low solubility and poor bioavailability, which ultimately limit their functionality and practical applications. Current approaches utilize bio-nanodelivery technologies to enhance polyphenol solubility, stability, sustained release, and targeting. Future research should focus on optimizing polyphenol delivery systems to enhance bioavailability and defining effective and safe dosage ranges for humans. Concurrently, precise polyphenol intake should be guided by the actual microbiota, as determined by metagenomics, across different physiological states. Conducting more high-quality clinical studies is essential to effectively bridge preclinical data with human health applications, advancing the practical value of polyphenols in disease prevention and health promotion.

## Figures and Tables

**Table 1:** Bioavailability of polyphenols.

Classification	Polyphenols	Structural Formula	Intake	Bioavailability	Reference
Flavonoids	Apigenin	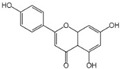	50 mg/kg	1.0%	[42]
	Luteolin	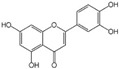	200 mg/kg	17.5%	[43]
	Quercetin	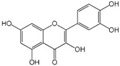	3 mg/mL	1.4%	[44]
	Kaempferol	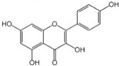	10 mg/kg	13.0%	[45]
	Myricetin	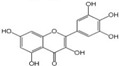	50 mg/kg	9.6%	[46]
	Naringenin	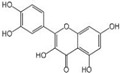	3.5 μg/mL	29.1%	[47]
	(-)-Catechin	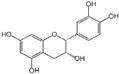	10 μmoL/L	8.0%	[48]
	Cyanidin	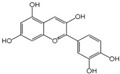	500 mg	12.4%	[49]
	Daidzein	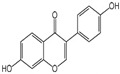	2 g/L	12.8%	[50]
Phenolic acids	Gallic acid	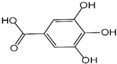	50 mg	1.1%	[51]
	Chlorogenic acid	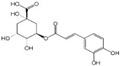	7.5 mg/L	20.0%	[52]
	Ferulic acid	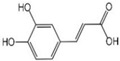	19 μg/kg	0.6%	[53]
	Caffeic acid	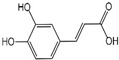	2 mg/kg	14.7%	[54]
Lignans	Sesamol	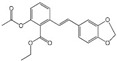	100 mg/kg	35.5%	[55]
	Pinoresinol	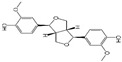	250 mg/L	9.1%	[56]
	Enterodiol	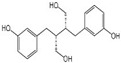	10 mg/kg	<1.0%	[57]
Stilbenes	Resveratrol	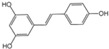	50 mg/kg	20.0%	[58]
	Pterostilbene	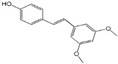	10 mg/kg	12.5%	[59]

## Data Availability

No new data were created or analysed in this study. Data sharing is not applicable to this article.
